# Percutaneous Coronary Intervention vs. Coronary Artery Bypass Grafting for Treating In-Stent Restenosis in Unprotected-Left Main: LM-DRAGON-Registry

**DOI:** 10.3389/fcvm.2022.849971

**Published:** 2022-04-29

**Authors:** Wojciech Wańha, Jacek Bil, Michalina Kołodziejczak, Adam Kowalówka, Mariusz Kowalewski, Damian Hudziak, Radosław Gocoł, Rafał Januszek, Tomasz Figatowski, Marek Milewski, Brunon Tomasiewicz, Piotr Kübler, Bruno Hrymniak, Piotr Desperak, Łukasz Kuźma, Krzysztof Milewski, Bartłomiej Góra, Andrzej Łoś, Jan Kulczycki, Adrian Włodarczak, Wojciech Skorupski, Marek Grygier, Maciej Lesiak, Fabrizio D'Ascenzo, Marek Andres, Paweł Kleczynski, Radosław Litwinowicz, Andrea Borin, Grzegorz Smolka, Krzysztof Reczuch, Marcin Gruchała, Robert J. Gil, Miłosz Jaguszewski, Krzysztof Bartuś, Piotr Suwalski, Sławomir Dobrzycki, Dariusz Dudek, Stanisław Bartuś, Mariusz Ga̧sior, Andrzej Ochała, Alexandra J. Lansky, Marek Deja, Jacek Legutko, Elvin Kedhi, Wojciech Wojakowski

**Affiliations:** ^1^Department of Cardiology and Structural Heart Diseases, Medical University of Silesia, Katowice, Poland; ^2^Department of Invasive Cardiology, Centre of Postgraduate Medical Education, Warsaw, Poland; ^3^Department of Anaesthesiology and Intensive Care, Ludwik Rydygier Collegium Medicum, Nicolaus Copernicus University, Antoni Jurasz University Hospital No. 1, Bydgoszcz, Poland; ^4^Yale University School of Medicine, New Haven, CT, United States; ^5^Department of Cardiac Surgery, Medical University of Silesia, Katowice, Poland; ^6^Department of Cardiac Surgery, Central Clinical Hospital of the Ministry of Interior, Centre of Postgraduate Medical Education, Warsaw, Poland; ^7^Thoracic Research Centre, Collegium Medicum, Nicolaus Copernicus University, Innovative Medical Forum, Bydgoszcz, Poland; ^8^Cardio-Thoracic Surgery Department, Heart and Vascular Centre, Maastricht University Medical Centre, Maastricht, Netherlands; ^9^Department of Cardiology, Jagiellonian University Medical College, Krakow, Poland; ^10^First Department of Cardiology, Medical University of Gdansk, Gdansk, Poland; ^11^Department of Heart Disease, Centre for Heart Disease, University Hospital Wroclaw, Wroclaw Medical University, Wrocław, Poland; ^12^Third Department of Cardiology, Medical University of Silesia, Zabrze, Poland; ^13^Department of Invasive Cardiology, Medical University of Bialystok, Białystok, Poland; ^14^Center for Cardiovascular Research and Development, American Heart of Poland, Katowice, Poland; ^15^Department of Cardiac and Vascular Surgery, Medical University of Gdansk, Gdansk, Poland; ^16^Department of Cardiology, Miedziowe Centrum Zdrowia, Lubin, Poland; ^17^Department of Cardiology, Poznan University of Medical Sciences, Poznań, Poland; ^18^Division of Cardiology, Department of Internal Medicine, Città della Salute e della Scienza, University of Turin, Turin, Italy; ^19^Department of Interventional Cardiology, Jagiellonian University Medical College Institute of Cardiology, John Paul II Hospital, Krakow, Poland; ^20^Department of Cardiovascular Surgery and Transplantology, Jagiellonian University, John Paul II Hospital, Krakow, Poland; ^21^Division of Cardiology, St-Jan Hospital, Brugge, Belgium

**Keywords:** left main, in-stent restenosis (ISR), coronary artery bypass graft (CABG), stents (Coronary), percutaneous coronary intervention (complex PCI)

## Abstract

**Background:**

Data regarding management of patients with unprotected left main coronary artery in-stent restenosis (LM-ISR) are scarce.

**Objectives:**

This study investigated the safety and effectiveness of percutaneous coronary intervention (PCI) vs. coronary artery bypass grafting (CABG) for the treatment of unprotected LM-ISR.

**Methods:**

Consecutive patients who underwent PCI or CABG for unprotected LM-ISR were enrolled. The primary endpoint was a composite of major adverse cardiac and cerebrovascular events (MACCE), defined as cardiac death, myocardial infarction (MI), target vessel revascularization (TVR), and stroke.

**Results:**

A total of 305 patients were enrolled, of which 203(66.6%) underwent PCI and 102(33.4%) underwent CABG. At 30-day follow-up, a lower risk of cardiac death was observed in the PCI group, compared with the CABG-treated group (2.1% vs. 7.1%, *HR* 3.48, 95%*CI* 1.01–11.8, *p* = 0.04). At a median of 3.5 years [interquartile range (*IQR*) 1.3–5.5] follow-up, MACCE occurred in 27.7% vs. 29.6% (*HR* 0.82, 95%*CI* 0.52–1.32, *p* = 0.43) in PCI- and CABG-treated patients, respectively. There were no significant differences between PCI and CABG in cardiac death (9.9% vs. 18.4%; *HR* 1.56, 95%*CI* 0.81–3.00, *p* = 0.18), MI (7.9% vs. 5.1%, *HR* 0.44, 95%*CI* 0.15–1.27, *p* = 0.13), or stroke (2.1% vs. 4.1%, *HR* 1.79, 95%*CI* 0.45–7.16, *p* = 0.41). TVR was more frequently needed in the PCI group (15.2% vs. 6.1%, HR 0.35, 95%*CI* 0.15–0.85, *p* = 0.02).

**Conclusions:**

This analysis of patients with LM-ISR revealed a lower incidence of cardiac death in PCI compared with CABG in short-term follow-up. During the long-term follow-up, no differences in MACCE were observed, but patients treated with CABG less often required TVR.

**Visual overview:**

A visual overview is available for this article.

**Registration:**

https://www.clinicaltrials.gov; Unique identifier: NCT04968977.

## Introduction

The left main coronary artery (LM) supplies a large myocardial area, therefore, atherosclerotic disease in the LM may lead to significant ischemia associated with high morbidity and mortality. Evidence from randomized controlled trials has shown that LM percutaneous coronary intervention (PCI) with drug-eluting stents (DES) is a feasible alternative to coronary artery bypass grafting (CABG) (1, 2); however, in-stent restenosis (ISR) after DES in unprotected LM disease continues to occur with an incidence of 9.7–17.6% (3, 4). A number of mechanical, biological, and technical factors predispose percutaneously revascularized patients to an increased risk of ISR. The use of intravascular imaging, proper stenting techniques, and calcium plaque modification improve outcomes of LM-PCI. Since LM-ISR can present as acute coronary syndrome (ACS) in substantial number of cases, treatment and decision-making process is often challenging. Although surgical revascularization is considered a standard treatment for this kind of stent failure, owing to a higher risk of perioperative morbidity and mortality, particularly in patients with high risk, as those with ACS, the restoration of flow with PCI may be a reliable alternative. The exact risk profile of unprotected patients with LM-ISR and variations of treatment choice remains a matter of an ongoing debate due to limited data in this clinical setting. Additionally, it is not clear whether repeat PCI is safe in these patients. Therefore, the purpose of the current study was to compare long-term outcomes following PCI or CABG for unprotected LM-ISR disease.

## Methods

The LM-DRAGON registry is a multi-center, observational study conducted in 16 high-volume centers in Poland and Italy between January 2000 and July 2020. Consecutive patients with LM-ISR defined as ≥50% diameter stenosis on angiography with or without multivessel coronary artery disease were included in the registry. Patients with LM distal bifurcation disease within the proximal 5 mm of the left anterior descending artery (LAD) or left circumflex artery (LCx) ostium (in the absence of significant angiographic stenosis in the LM) were also eligible (LM equivalent). Patients with protected LM-ISR, defined as the occurrence of ≥1 patent arterial or venous graft to the left coronary artery, or other concomitant non-CABG procedure during surgery were excluded.

The choice of the type of revascularization (PCI or CABG) was at the discretion of heart team or individual invasive cardiologist, if the patient was unstable (acute LM occlusion). The choice of techniques for LM PCI or CABG was at the operator's discretion as well. The 4-stage classification (5) was used to determine the degree of restenosis on the basis of restenosis in relation to stented length based on the angiographic manifestation: (i) focal (≤ 10 mm length); (ii) diffuse (>10 mm within the stent); (iii) proliferative (>10 mm extending outside the stent); and (iv) occlusive ISR. Angiographic visual estimation or intravascular imaging was used to diagnose LM restenosis. Significant stenosis was defined as intravascular ultrasound (IVUS) imaging of the target lesion with a minimum lumen area (MLA) of ≤ 6 mm^2^ for the left main lesions was defined as significant stenosis. Angiographic data of patients included in the study were collected and recorded in the central cardiovascular information registry. Bifurcation lesions were classified according to the Medina classification (6). The European Bifurcation Club consensus document was used to define the one or two stent strategy of LM PCI (7). Patient data were anonymized in each center, combined into a database, and statistically analyzed as a single cohort. The institutional review board at each center approved the study protocol; however, due to the retrospective nature of the study, no written informed consent was needed. The patient data were protected according to the requirements of country law and hospital standard operating procedures. The data that support the findings of this study are available from the corresponding author upon reasonable request. The study was conducted in accordance with the Declaration of Helsinki and was registered at ClinicalTrials.gov (NCT04968977).

### Endpoints

The primary endpoint was a composite of major adverse cardiac and cerebrovascular events (MACCE), defined as cardiac death, myocardial infarction (MI), target vessel revascularization (TVR), or stroke assessed during a median of 3.5 year follow-up [interquartile range (*IQR*) 1.3–5.5]. TVR was defined as any repeat intervention (PCI or CABG) of the treated vessel caused by ischemia driven stenosis of the LM. Data regarding long-term outcomes were obtained by phone call or clinical visit as well as from the National Health Fund Service (Ministry of Health) database.

### Statistical Analysis

Continuous data are presented as mean ± standard deviation or median with IQR (Q1–Q3). Categorical data are expressed as count and percentage. Normal distribution was verified by the Kolmogorov–Smirnov test. Continuous data were compared by the Student *t*-test or by Mann–Whitney *U* test, depending on the data distribution. Categorical data were analyzed with the χ^2^ or Fisher exact test. Kaplan–Meier survival curves were performed to present the unadjusted time-to-event data for investigated endpoints and were compared using the log-rank test. Finally, Cox regression for 30 days, 1 year, and long-term follow-up event rates of MACCE, cardiac death, TLR, TVR, MI, and stroke were calculated for both groups. A *p*-value <0.05 was considered statistically significant. The statistical analysis was performed using MedCalc version 17.9.2 (MedCalc Software, Ostend, Belgium) and SPSS version 21 (IBM Corp, Armonk, NY).

## Results

The LM-DRAGON registry included 305 patients, of whom 203 (66.6%) were treated with PCI and 102 (33.4%) with CABG (Table 1). After verifying missing outcomes with multiple datasets, 12 (5.9%) patients in the PCI group and 4 (3.9%) in the CABG group were lost to follow-up. A comparison between PCI and CABG groups demonstrated significant differences in baseline characteristics and clinical presentation. Patients treated by PCI were older (68.9 ± 10.3 vs. 65.0 ± 8.9, *p* < 0.001) more often had diabetes mellitus (49.8% vs. 35.3%, *p* = 0.02), and chronic kidney disease (25.6% vs. 13.7%, *p* = 0.02), compared with CABG patients. STS score for mortality and morbidity was lower in the PCI group [4.5 (*IQR* 2.5–8.4) vs. 7.2 (*IQR* 5.1–9.9), *p* < 0.001]; however, there were no differences in EuroSCORE II [1.5 (0.9–3.5) vs. 1.6 (1.0–3.3), *p* = 0.52].

**Table 1:** Patient characteristics, risk factors, and clinical presentation according to the type of treatment.

	**PCI** **(*n* = 203)**	**CABG** **(*n* = 102)**	***p*-value**
Age, y	68.9 ± 10.3	65.0 ± 8.9	<0.001
Male sex	148 (72.9)	72 (70.6)	0.67
Body mass index, kg/m^2^	28.4 ± 3.9	27.7 ± 3.7	0.22
Discharge diagnosis			
Chronic coronary syndrome, n (%)	80 (39.4)	19 (18.6)	<0.001
Unstable angina, n (%)	46 (22.7)	62 (60.8)	<0.001
Non–ST-segment elevation myocardial infarction	72 (35.5)	21 (20.6)	0.007
ST-segment elevation myocardial infarction	4 (2.0)	0 (0)	0.15
Previous myocardial infarction	134 (66.0)	65 (63.7)	0.69
Previous CABG	33 (16.3)	1 (1.0)	<0.001
Previous stroke	15 (7.4)	4 (3.9)	0.24
Diabetes mellitus	101 (49.8)	36 (35.3)	0.02
Insulin requiring	35 (17.2)	19 (18.6)	0.77
Hypertension	170 (83.7)	92 (90.2)	0.13
Hyperlipidemia	167 (82.3)	85 (83.3)	0.82
Chronic kidney disease^*^	52 (25.6)	14 (13.7)	0.02
Dialysis	3 (1.5)	2 (2.0)	0.75
Atrial fibrillation	29 (14.3)	13 (12.7)	0.71
Current smoker	30 (14.8)	16 (15.7)	0.83
Family history of coronary artery disease	35 (17.2)	22 (21.6)	0.36
Pulmonary disease	24 (11.8)	2 (2.0)	0.003
Peripheral artery disease	46 (22.7)	16 (15.7)	0.15
Cardiac arrest before PCI/CABG	9 (4.4)	1 (1.0)	0.11
Time to restenosis, months	10.0 (5.0–19.0)	6.5 (4.0–33.0)	0.22
Recurrent in-stent restenosis	42 (20.7)	10 (9.8)	0.02
Number of in-stent restenosis events	1.2 ± 0.4	1.1 ± 0.4	0.03
STS score mortality and morbidity	4.5 (2.5–8.4)	7.2 (5.1–9.9)	<0.001
EuroSCORE II	1.5 (0.9–3.5)	1.6 (1.0–3.3)	0.52
Left ventricular ejection fraction, %	50.0 (40.0–60.0)	49.0 (40.0–55.0)	0.46

*Values are mean ± standard deviation, n (%), or median (interquartile range).*

* *Estimated glomerular filtration rate of <60 ml/min/1.73 m^2^ calculated using the modification of diet in renal disease method. CABG, coronary artery bypass grafting; PCI, percutaneous coronary intervention; STS, society of thoracic surgeons*.

Angiographic, procedural, and medication data are shown in Table 2. The SYNTAX score I did not differ between the two groups [22.0 (13.2–27.0) vs. 21.5 (15.0–27.0), *p* = 0.47]. Recurrent ISR was more common in the PCI group (20.7% vs. 9.8%, *p* = 0.02). Procedurally, the most common location of LM-ISR was the distal segment including the bifurcation. True bifurcation lesions (Medina 1,1,1) were more prevalent in the PCI, compared with the CABG group (47.0% vs. 28.6%, *p* < 0.001). Patients treated with PCI had a higher prevalence of focal ISR (55.7% vs. 39.2%, *p* = 0.02) and proliferative ISR (12.8% vs. 11.8%, *p* = 0.02), while those in the CABG group had a higher prevalence of diffuse ISR (31.0% vs. 49.0%, *p* = 0.02). Number of stent layers in the target segment was higher in PCI (1.2 ±0.4 vs. 1.0 ± 0.2, *p* < 0.001). In the PCI group, 59.6% of patients underwent DES implantation, 38.4% were treated with a drug coated balloon, and 2% were treated with plain old balloon angioplasty; additionally, 3 patients had intravascular lithotripsy during PCI. TIMI 3 flow post-PCI was observed in 98.5% of patients and residual stenosis was observed in 8.9%. In the CABG group, 90.2% patients had left internal mammary artery to left anterior descending grafts, 9.8% had vein to left anterior descending grafts, and 67.6% had grafts to obtuse marginal branch or distal Cx. Periprocedural mechanical circulatory support was needed more often in the CABG group (7.8% vs. 2.0%, *p* = 0.02).

**Table 2:** Angiographic, procedural, and medication data according to the type of treatment.

	**PCI** **(*n* = 203)**	**CABG** **(*n* = 102)**	***p*-value**
Restenosis in drug-eluting stents	185 (91.1)	78 (76.5)	<0.001
Restenosis in bare metal stents	18 (8.9)	24 (23.5)	
SYNTAX score I	22.0 (13.2–27.0)	21.5 (15.0–27.0)	0.47
SYNTAX score II (PCI)	32.5 (22.4–44.8)	32.5 (25.9–41.6)	0.75
SYNTAX score II (CABG)	39.2 (24.7–48.6)	29.1 (21.7–37.0)	<0.001
Number of diseased vessels			
1	31 (15.3)	9 (8.8)	0.12
2	76 (37.4)	28 (27.5)	0.08
3	96 (47.3)	65 (63.7)	0.006
Previous left main PCI strategy			
One-stent strategy	157 (77.3)	70 (68.6)	0.10
Two-stent strategy	46 (22.7)	32 (31.4)	
In-stent restenosis left main segment			
Proximal/medial	18 (8.9)	4 (3.9)	0.12
Distal	185 (91.1)	98 (96.1)	
Medina classification			
1,1,1	87 (47.0)	28 (28.6)	<0.001
1,1,0	23 (12.4)	34 (34.7)	
1,0,1	41 (22.2)	13 (13.3)	
0,1,1	8 (4.3)	11 (11.2)	
1,0,0	8 (4.3)	10 (10.2)	
0,1,0	13 (7.0)	2 (2.0)	
0,0,1	5 (2.7)	0 (0)	
Type of in-stent restenosis			
Focal	113 (55.7)	40 (39.2)	0.02
Diffuse	63 (31.0)	50 (49.0)	
Proliferative	26 (12.8)	12 (11.8)	
Occlusive	1 (0.5)	(0)	
Restenotic stent length, mm	18.0 (16.0–23.0)	22.2 (18.0–27.0)	0.11
Restenotic stent diameter, mm	3.5 (3.5–4.0)	3.5 (3.5–4.0)	0.82
Thrombus	3 (1.5)	(0)	0.26
Stenosis, %	70.0 (60.0–90.0)	90.0 (80.0–90.0)	<0.001
Number of stent layers	1.2 ± 0.4	1.0 ± 0.2	<0.001
PCI data			
PCI with drug-eluting stents^*^	121 (59.6)	—	—
PCI with drug-coated balloon	78 (38.4)	—	—
Plain old balloon angioplasty	4 (2.0)	—	—
Intravascular lithotripsy	3 (1.5)	—	—
Procedural use of intracoronary imaging	81 (39.9)	—	—
Residual stenosis	18 (8.9)	—	—
TIMI 3 post-PCI	199 (98.5)	—	—
Perforation	1 (0.5)	—	—
Dissection	1 (0.5)	—	—
Stent thrombosis during hospitalization	1 (0.5)	—	—
Complications during PCI	15 (7.4)	—	—
CABG data			
Off-pump coronary artery bypass	—	16 (15.7)	—
Minimally invasive coronary artery bypass	—	1 (1.0)	—
Left internal mammary artery to LAD	—	92 (90.2)	—
Aorta to LAD	—	10 (9.8)	—
Aorta to obtuse marginal	—	69 (67.6)	—
Aorta to right coronary artery	—	40 (39.2)	—
Arterial grafts	-	0.9 ± 0.3	—
Vein grafts	-	1.2 ± 0.7	—
CABG–other type	—	11 (10.8)	—
Complications during CABG, n (%)	—	15 (16.1)	—
Complete revascularization, n (%)	—	91 (89.2)	—
Mechanical circulatory support	4 (2.0)	6 (7.8)	0.02
Glycoprotein IIb/IIIa inhibitors	9 (4.4)	(0)	—

*Values are mean ± standard deviation, n (%), or median (interquartile range). CABG, coronary artery bypass grafting; LA, left anterior descending artery; PCI, percutaneous coronary intervention; TIMI, thrombolysis in myocardial infarction;*

* *all drug eluting stents were 2nd generation*.

### 30 Days and 1-Year Outcomes

At 30-day follow-up, there was a lower risk of cardiac death in the PCI group (2.1% vs. 7.1%, *HR* 3.48, 95% *CI* 1.01–11.8, *p* = 0.04) as compared to CABG treatment group. However, worth mentioning, patients who died in CABG group were at median EuroSCORE II 3.4 (2.3–4.5) and median STS score for mortality and morbidity 9.4 (8.6–11.4). There were no differences with respect to MACCE (3.1% vs. 7.1%, *HR* 2.32, 95% *CI* 0.77–6.90, *p* = 0.13), TVR (PCI−0.5% vs. CABG−0%), MI (PCI−0% vs. CABG−0%), and stroke (PCI−0.5% vs. CABG−0%) through 30-days. During 1-year follow-up a trend toward a higher rate of TVR in the PCI group (7.9% vs. 2.0%; *HR* 0.25, 95% *CI* 0.05–1.09, *p* = 0.07) was observed, with no differences in MI (3.7% vs. 2.0%, *HR* 0.54, 95% *CI* 0.11–2.60, *p* = 0.44), cardiac death (4.2% vs. 8.2%, *HR* 1.98, 95% *CI* 0.74–5.27, *p* = 0.17), stroke (1.6% vs. 1.0%, *HR* 0.64, 95% *CI* 0.06–6.16, *p* = 0.70) and MACCE (14.7% vs. 12.2%, *HR* 0.81, 95% *CI* 0.41–1.59, *p* = 0.54) (Table 3).

**Table 3:** One-year and long-term follow-up according to the type of treatment.

	**30 days follow-up**				**1-year follow-up**				Long-Term follow-up^*^			
	**PCI**	**CABG**	***HR* (95%*CI*)**	***p*-value**	**PCI**	**CABG**	***HR* (95%*CI*)**	***p*-value**	**PCI**	**CABG**	***HR* (95%*CI*)**	***p*-value**
**TVR**	1 (0.5)	-	-	-	15 (7.9)	2 (2.0)	0.25 (0.05–1.09)	0.07	29 (15.2)	6 (6.1)	0.35 (0.15–0.85)	0.02
**MI**	-	-	-	-	7 (3.7)	2 (2.0)	0.54 (0.11–2.60)	0.44	15 (7.9)	5 (5.1)	0.44 (0.15–1.27)	0.13
**Cardiac death**	4 (2.1)	7(7.1)	3.48 (1.01–11.8)	0.04	8 (4.2)	8 (8.2)	1.98 (0.74–5.27)	0.17	19 (9.9)	18 (18.4)	1.56 (0.81–3.00)	0.18
**Stroke**	1(0.5)	-	-	-	3 (1.6)	1 (1.0)	0.64 (0.06–6.16)	0.70	4 (2.1)	4 (4.1)	1.79 (0.45–7.16)	0.41
**MACCE**	6 (3.1)	7 (7.1)	2.32 (0.77–6.90)	0.13	28 (14.7)	12 (12.2)	0.81 (0.41–1.59)	0.54	53 (27.7)	29 (29.6)	0.82 (0.52–1.32)	0.43

* *PCI median follow-up period 3.4 (IQR 1.3–5.2) years; CABG median follow-up period 3.8 (IQR 2.3–6.5) years.*

*CABG, coronary artery bypass grafting; CI, confidence intervals; HR, hazard ratio; MACCE, major adverse cardiac and cerebrovascular events; MI, myocardial infarction; PCI, percutaneous coronary intervention; TVR, target vessel revascularization*.

### Long-Term Outcomes

**Figure 1:**
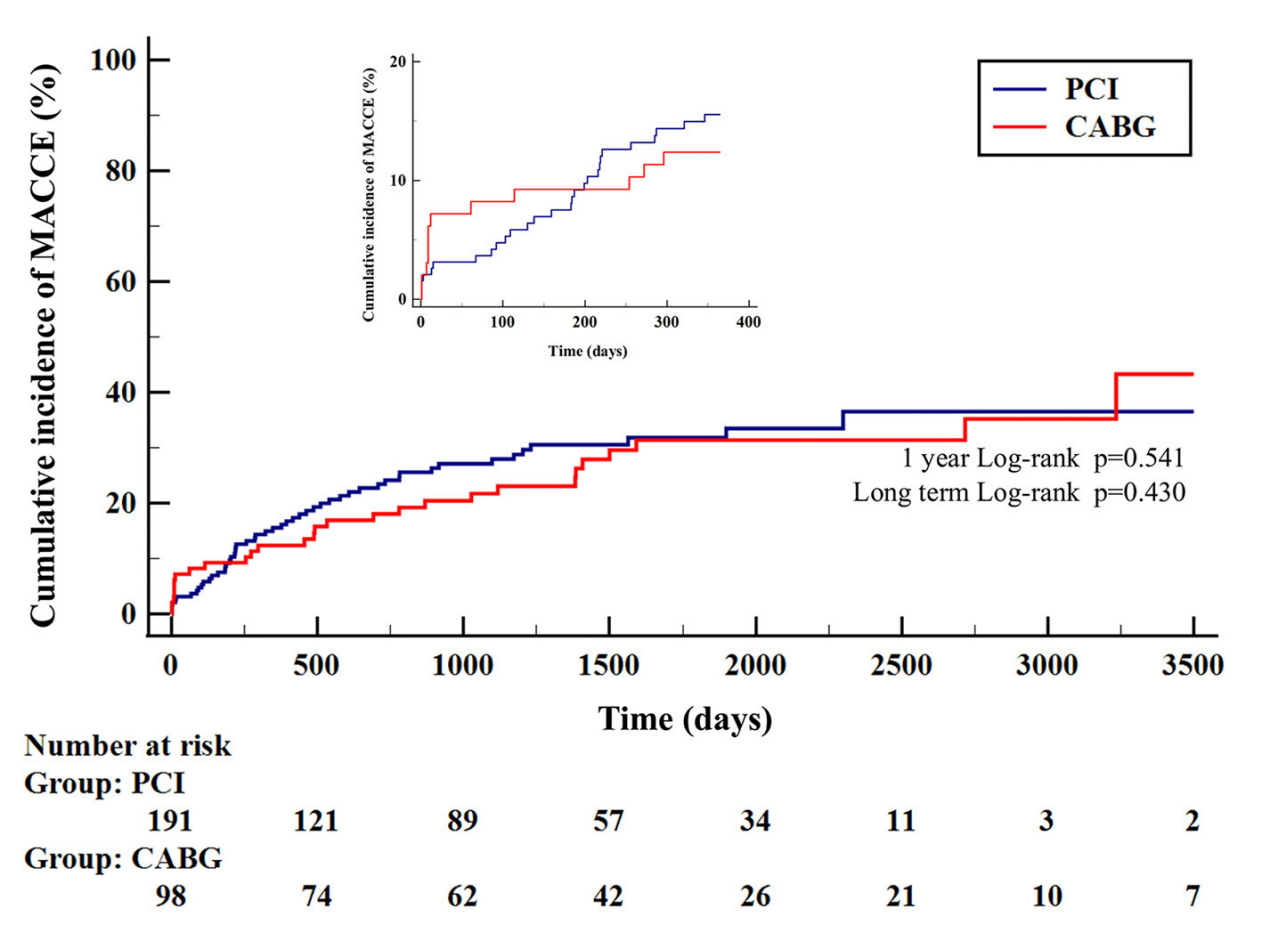
Kaplan–Meier curves for MACCE according to type of treatment. Major adverse cardiac and cerebrovascular events (MACCE) is the composite of target vessel revascularization, myocardial infarction, stroke, or cardiac death. CABG, coronary artery bypass grafting; PCI, percutaneous coronary intervention.

**Figure 2:**
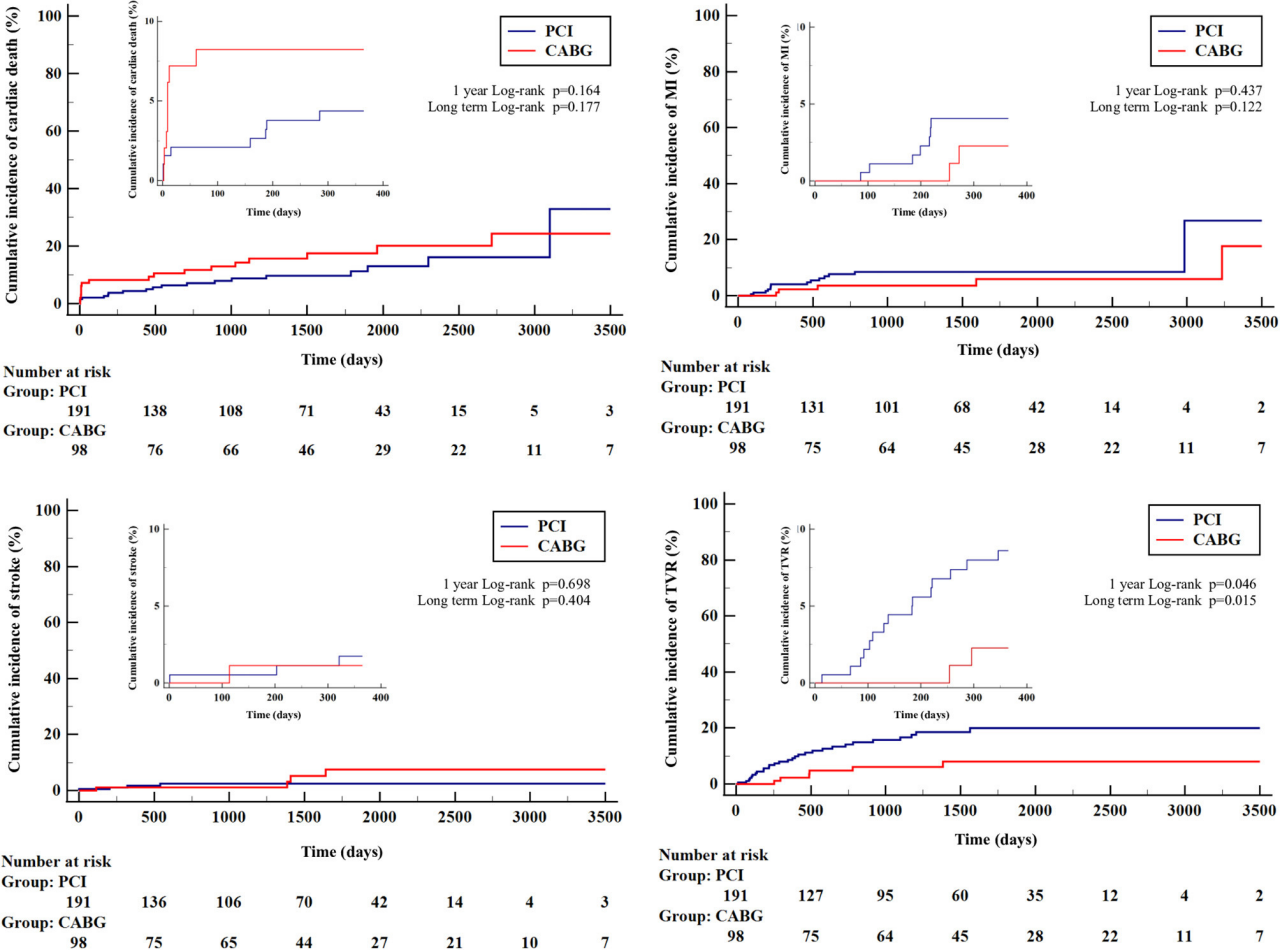
Kaplan–Meier curves for cumulative incidence of secondary outcomes according to type of treatment. CABG, coronary artery bypass grafting; MI, myocardial infarction; PCI, percutaneous coronary intervention; TVR, target vessel revascularization.

**Figure 3:**
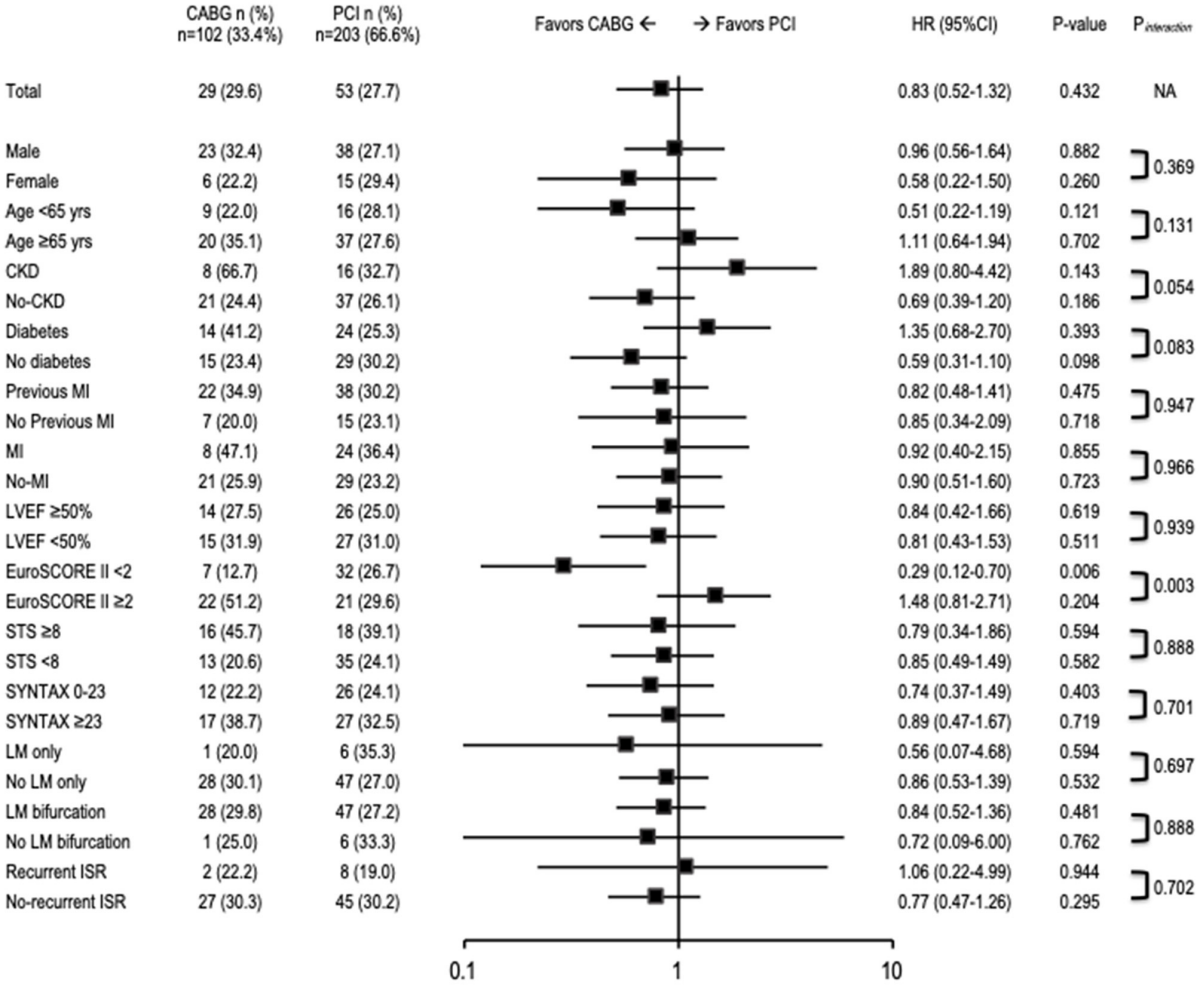
Risk of MACCE at long-term follow-up. CABG, coronary artery bypass grafting; CI, confidence interval; CKD, chronic kidney disease; HR, hazard ratio; ISR, in-stent restenosis; LM, left main coronary artery; LVEF, left ventricular ejection fraction; MI, myocardial infarction; PCI, percutaneous coronary intervention; STS, Society of Thoracic Surgeons.

The median follow-up period was 3.4 years (1.3–5.2) in the PCI group and 3.8 years (2.3–6.5) in the CABG group (*p* = 0.046). The study's primary endpoint occurred in 27.7% of patients in PCI group and 29.6% of patients in CABG group (*HR* 0.82, 95% *CI* 0.52–1.32, *p* = 0.43) (Table 3). There were no significant differences between PCI and CABG in terms of cardiac death (9.9% vs. 18.4%; *HR* 1.56, 95% *CI* 0.81–3.00, *p* = 0.18), MI (7.9% vs. 5.1%; *HR* 0.44, 95% *CI* 0.15–1.27, *p* = 0.13), or stroke (2.1% vs. 4.1%; *HR* 1.79, 95% *CI* 0.45–7.16, *p* = 0.41); however, TVR occurred less frequently in the CABG group than in the PCI group (6.1% vs. 15.2%, *HR* 0.35, 95% *CI* 0.15–0.85, *p* = 0.02). The treatment strategy of TVR after PCI and CABG is reported in Supplementary Material. Kaplan–Meier curves for the cumulative incidence of selected outcomes are shown in Figures 1, 2. The results of the combined clinical outcome measures and MACCE were consistent across most of the pre-specified subgroups (Figure 3). Patients at lower preoperative risk (EuroSCORE II <2) had significantly less MACCE in the CABG group than in the PCI group.

## Discussion

We present the largest registry of patients with unprotected LM-ISR reporting long-term data on the safety and efficacy of revascularization with either PCI or CABG. In the current report, both PCI and CABG provided favorable clinical outcomes; however, a lower incidence of cardiac death at 30-day follow-up was observed in the PCI group compared with the CABG group. This was reflected in the subgroup analysis, where high EuroSCORE II favored PCI treatment. The elevated risk of the patients with CABG treatment was also indicated by a substantial proportion of mechanical circulatory support use. Clinically compromised patients characterized by such a procedural profile could therefore drive the short-term excess mortality in the CABG-revascularized group. At the long-term follow-up patients receiving PCI treatment, compared with those treated with CABG, had similar rates of cardiac death but a higher rate of TVR. Our long-term results provide evidence for the use of PCI in unprotected LM-ISR and suggest its safety and efficacy in reducing recurrent stent failure.

Despite favoring results, LM-ISR PCI is, undoubtedly, a challenging treatment option. Those with LM-ISR are a specific subset of patients who already underwent high-risk procedure of PCI in LM and now experience a subsequent stent failure. Previous reports addressed a combination of multiple factors contributing to an increased risk of LM-ISR and the subsequent adverse events: female sex, a previous restenotic lesion, a total number of stents employed, distal bifurcation lesions, and the use of complex bifurcation stenting technique (4), whereas the use of IVUS was protective (8). To systematically apprise the phenomenon, ISR classification including variables contributing to in different angiographic manifestation of ISR lesion length and the location of the neointimal proliferation, was proposed (9). To date, many large-scale clinical studies have evaluated treatment strategies for patients with *de novo* unprotected LM disease. Generally, guidelines recommend CABG revascularization in patients with *de novo* unprotected LM disease with high SYNTAX scores, downplaying the role of PCI (10). Although the less invasive PCI has a lower rate of periprocedural adverse events and provides more rapid recovery compared with CABG (11), it exposes patients to an increased risk of myocardial ischemia in LM-ISR. A previous study demonstrated that DES implantation or drug-coated balloon angioplasty could be effective in patients with ISR (12, 13); however, the effectiveness of repeat PCI for LM-ISR following previous DES implantation remains controversial. The Milan and New-Tokyo (MITO) registry evaluated the prognostic role of restenosis in unprotected distal LM bifurcation coronary lesions and revealed that the patients with LM main branch ISR have higher risk of cardiac mortality compared with patients without LM main branch ISR (14). As limited data are available on the LM-ISR optimal revascularization, this clinical setting remains a matter of discussion. The Failure in Left Main Study (FAILS) study showed satisfactory results using PCI revascularization strategy at 27 months of follow-up, with major adverse cardiac events (MACE; death, MI, or TLR) occurring in 26% of patients and TLR occurring in 22%; however, the analyzed groups were too small to allow for a comparison between the two treatment strategies (3). Promising results of PCI were also reported in the long-term results of the CORPAL registry, where few patients were treated by CABG over the course of 46 ± 26 months (15). The rate of outcomes in PCI patients was 22% MACE (cardiac death, TLR, and MI), 8% cardiac death, 4% non-fatal MI, and 15% repeat revascularization. The optimal management of patients with LM-ISR focuses on maintaining a balance between the long-term risk of TVR in PCI and perioperative complications in CABG; however, the PCI in LM-ISR is oftentimes performed as a first-line, life-saving treatment in unstable patients with acute LM occlusion compared with emergency cardiac surgery. Safety and efficacy of both revascularization methods were evaluated in many studies in *de novo* unprotected LM lesions, showing a comparable rate of clinical outcomes in terms of MACCE (1, 11, 16). Long-term results of the LE MANS, PRECOMBAT, and EXCEL trials showed that at 1-year and 5-year follow-up, patients undergoing revascularization for unprotected LM experienced similar rate of the composite clinical outcome. The rate of target vessel failure in the LE MANS and the rate of mortality, MI, and stroke in PRECOMBAT were also comparable between PCI and CABG (11, 16). The results of TVR varied between studies, with a hint of more frequent occurrence in the PCI vs. CABG, also observed in the current LM-DRAGON registry. None of the previous randomized controlled trials directly compared PCI and CABG for reintervention for ISR in LM lesions; indeed, ISR or prior LM intervention has universally been imposed as exclusion criterion in these trials (17).

### Limitations

There are several limitations to this study. First, we had no intravascular imaging data and thus limited insight into the mechanisms of restenosis. We had no comprehensively reported data on initial PCI strategy, nor on completeness of revascularization in the PCI group. Angiographic follow-up was not systematically performed. In the PCI group, 16% of patients had previous CABG, which may also affect further revascularization options, furthermore the decisions on the choice of treatment were not random but based on the heart team or operator's preference; selection bias was inevitable and may limit our interpretation. The study was a retrospective analysis with inherent limitations; however, this was balanced by an “all-comer” design with broad inclusion criteria and a large sample size.

## Conclusions

This analysis of a real-life unprotected LM-ISR registry revealed a lower incidence of cardiac death in the PCI treatment group compared with the CABG treatment group at short-term follow-up. Long-term follow-up showed similar incidences of cardiac death, MACCE, MI, and stroke regardless of revascularization strategy, but patients who underwent CABG less often required TVR compared with patients who underwent PCI.

## Data Availability Statement

The original contributions presented in the study are included in the article/Supplementary Material, further inquiries can be directed to the corresponding authors.

## Ethics Statement

The studies involving human participants were reviewed and approved by Medical University of Silesia, Katowice, Poland. Written informed consent for participation was not required for this study in accordance with the national legislation and the institutional requirements.

## Author Contributions

Data curation: JB, AK, DH, RG, RJ, TF, MM, BT, PKÜ, PD, ŁK, KM, BG, AŁ, JK, AW, MA, PKL, RL, AB, and GS. Formal analysis and methodology: WWA, MKOŁ, and MKOW. Supervision: WWA, WWO, MG, ML, FD'A, MA, KR, MGR, RG, MJ, KB, PS, SD, DD, SB, MGA̧, JL, AO, AL, MD, and EK. Writing—original draft: WWA and MKOŁ. All authors have read and agreed to the published version of the manuscript.

## Conflict of Interest

The authors declare that the research was conducted in the absence of any commercial or financial relationships that could be construed as a potential conflict of interest.

## Publisher's Note

All claims expressed in this article are solely those of the authors and do not necessarily represent those of their affiliated organizations, or those of the publisher, the editors and the reviewers. Any product that may be evaluated in this article, or claim that may be made by its manufacturer, is not guaranteed or endorsed by the publisher.
