# Supplementary material for: Percutaneous Coronary Intervention vs. Coronary Artery Bypass Grafting for Treating In-Stent Restenosis in Unprotected-Left Main: LM-DRAGON-Registry
Source: Front Cardiovasc Med. 2022 Apr 29;9:849971. doi: 10.3389/fcvm.2022.849971 (PMC9125786; doi:10.3389/fcvm.2022.849971)
Supplement: Supplementary file 1 [file Table_1.DOCX]

| Supplement 1. Treatment strategy of TVR after PCI and CABG | | |
| --- | --- | --- |
|  | PCI | CABG |
| TVR post PCI | 25 (86.2) | 4 (13.8) |
| TVR post CABG | 5 (83.3) | 1 (16.7) |
| Values are n (%)  CABG = coronary artery bypass grafting; PCI = percutaneous coronary intervention; TVR = target vessel revascularization | | |
