# Supplementary figures and images for: Percutaneous Coronary Intervention vs. Coronary Artery Bypass Grafting for Treating In-Stent Restenosis in Unprotected-Left Main: LM-DRAGON-Registry
Source: Front Cardiovasc Med. 2022 Apr 29;9:849971. doi: 10.3389/fcvm.2022.849971 (PMC9125786; doi:10.3389/fcvm.2022.849971)

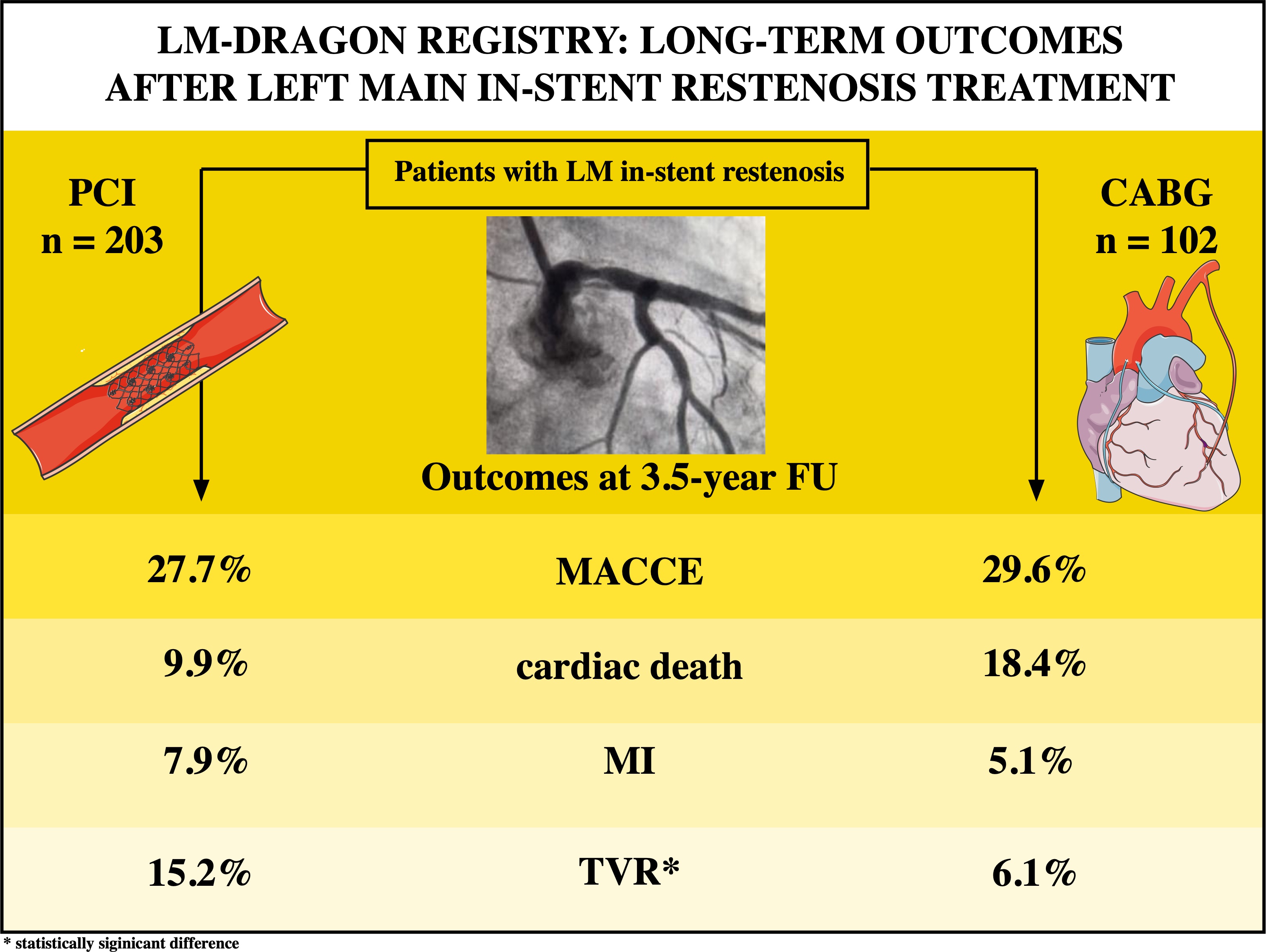

Supplement: Supplementary file 2 [file Image_1.jpg]
